# Agar oligosaccharides ameliorate the intestinal inflammation of male *Drosophila melanogaster* via modulating the microbiota, and immune and cell autophagy

**DOI:** 10.1002/fsn3.2108

**Published:** 2021-01-08

**Authors:** Chao Ma, Yifan Wang, Guocai Zhang, Xianjun Dai

**Affiliations:** ^1^ College of Life Sciences China Jiliang University Hangzhou, Zhejiang China

**Keywords:** agar oligosaccharides, autophagy, *Drosophila*, intestinal inflammation, microbiota

## Abstract

Agar oligosaccharide (AOS) is a marine prebiotic with apparent improving health and longevity effects. In this study, the protective effect of AOS on the intestine was evaluated in the sodium dodecyl sulfate (SDS)‐induced inflammatory model of male *Drosophila*. The results showed that AOS used as a nutritional additive in basal food could lengthen the life of SDS‐stimulated male *Drosophila*. Additionally, AOS could alleviate the injuries of SDS to microvilli and mitochondria in male *Drosophila* midgut epithelial cells. AOS could regulate the relative gene expressions in the antibacterial peptides (AMPs), mTOR pathway and autophagy process, and significantly improved the α‐diversity of midgut microbiota and decreased the abundance of *Klebsiella aerogenes,* a kind of bacteria easily causing infections. Collectively, AOS could ameliorate the intestinal inflammation by modulating the microbiota, and the gene expression of immune and cell autophagy.

## Graphical abstract

AOS could alleviate the injuries of SDS to microvilli and mitochondria in male Drosophila midgut epithelial cells. AOS improved the α‐diversity of midgut microbiota and decreased the abundance of *Klebsiella aerogenes*. AOS could ameliorate the intestinal inflammation by modulating the microbiota, and the gene expression of immune and cell autophagy.

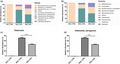

## 1 INTRODUCTION

The intestine, when digesting and absorbing nutrients, creates an important barrier between the internal and external environments of the organism. The intestinal mucosa is continuously exposed to many antigens produced by ingested food, bacteria, and invading viruses. When the antigen penetrates through the epithelial layer, it may cause abnormal immune stimulation (Söderholm & Perdue, 2001). Studies have shown that the integrity of the intestine is beneficial to the health of the host, and the intestine can serve as a signal transmission center for the rate of cellular senescence (Rera et al., 2013; Suo et al., 2017). Intestinal epithelial cells, especially those of young animals, are vulnerable to inflammation and infection, which was proven in pigs (Xu et al., 2018). Inflammation often results in intestinal mucosal damage and barrier function impairment, which is linked to multiple markers of aging in male *Drosophila*, including systemic metabolic dysfunction, increasing expression of immunity‐related genes, and reducing spontaneous physical activity (Rera et al., 2012).

In addition to the above physiological injuries, inflammation stress could be directly related to the microbiota, immune system, and cell autophagy in the intestine. Recently, it was reported that the alterations of intestinal microbiota could be associated with age‐onset barrier dysfunction and aging of the host (Clark et al., 2015). Integrity of intestinal barrier function is increasingly linked to gut microbiota, diet, and innate immunity (Chakaroun et al., 2020). By influencing the intestinal gene expression and microbial composition, dietary factors could affect the health of *Drosophila* via modulating gut health and intestinal epithelial integrity (Biteau et al., 2008). Additionally, as an evolutionary conserved catabolic and homeostatic process, autophagy could be cytoprotective and tissue‐protective by clearing deleterious and unnecessary cytosolic components (Jacomin & Nezis, 2020). Autophagy was shown to provide a large enough energy supply in a stress reaction (Chang & Neufeld, 2010).

In recent years, several reports showed that the nutritional additive could prevent and alleviate chronic diseases, which have received increasingly attention. For example, *Lycium ruthenicum* Murray ethanol extract could prevent and attenuate inflammatory bowel diseases in dextran sulfate sodium‐induced (DSS) murine experimental colitis (Zong et al., 2020), and curcumin could protect the brain, liver, and kidneys from oxidative damage (Samarghandian et al., 2017). Agar oligosaccharides (AOSs), as a marine prebiotic, have the repeating agarobiose units composed of D‐galactose at the nonreducing and 3,6‐anhydro‐L‐galactose at the reducing ends (Higashimura et al., 2013). Studies have shown that AOSs have an inhibiting effect on murine intestinal inflammation through the induction of heme oxygenase‐1 expression and also indicate many immunological effects through the suppression of elevated levels of nitric oxide, prostaglandin E(2), and pro‐inflammatory cytokines, such as tumor necrosis factor‐α, interleukin‐1β, and interleukin‐6 in lipopolysaccharide‐stimulated monocytes and macrophages (Enoki et al., 2010; Higashimura et al., 2013). Therefore, AOS could be used as a nutritional additive in the food to prevent inflammatory diseases of the intestine. But there are no reports about AOS affecting the microbiota, cell pathways, and autophagy in the inflammatory intestine, which could elucidate the underlying mechanism. In this study, male *Drosophila* were used as the research object to confirm the anti‐inflammatory effects of AOS in vivo via modulating the microbiota, and immune and cell autophagy.

## 2 MATERIALS AND METHODS

### 2.1 Experimental sample

Pharmaceutical‐grade AOS (≥95%) was purchased from Qingdao Bozhi Huili Biotechnology Co., Ltd., China. It was obtained by acid hydrolysis of the agar, which was mainly composed of agarobiose (A2), agarotetraose (A4), and agarohexaose (A6) (Wang, 2019). AOS was dissolved in sterile water and passed through a 0.22‐μm microporous filter to prepare a sterile aqueous solution.

### 2.2 *Drosophila* culture

The Canton S lines of *Drosophila melanogaster* were obtained from the *Drosophila* Stock Center at the Shanghai Academy of Life Sciences, Chinese Academy of Sciences. The *Drosophila* was raised at 24 ± 1°C under 55% relative humidity with a 12/12‐hr light/dark cycle. Based on the results of a previous study, the newly emerged male fruit flies (within 8 hr) were randomly divided under carbon dioxide gas (CO_2_) into control and experimental groups (the *Drosophila* mentioned in the following text is referred to male *Drosophila*). In the control group, *Drosophila* was cultured on a basal diet–yeast medium, and the *Drosophila* in the experimental group was fed a basal diet supplemented with 0.125% AOS. All other conditions were consistent between the groups.

### 2.3 SDS challenge assay

At the fifth day of above culture, the flies of two groups from the basal diet–yeast medium were, respectively, fed on two solutions, one containing 5% sucrose (SUC_CTRL group) and the other containing 5% sucrose added with 0.6% SDS (SDS_CTRL group), and the flies from the basal diet supplemented with 0.125% AOS were fed on the solution containing 5% sucrose, 0.6% SDS, and 0.125% AOS (SDS_AOS group) (Zhang et al., 2020). The each group included four biological replicates. The operating procedure was as follows: 15 flies as a biological replicate were removed into a tube to be fasted for 2 hr and then transferred to a new vial containing filter paper impregnated with the above solution. The number of *Drosophila* deaths in each vial was counted every 12 hr until all *Drosophila* death. The significance of survival curve differences was analyzed using the log‐rank (Mantel–Cox) test with GraphPad Prism 6 (Version No. 6.01; GraphPad Software).

### 2.4 Ultrastructural examination of epithelial cells in *Drosophila* midgut

Ten surviving *Drosophila* from each group were randomly taken from samples at the 96th hour after the induction, and firstly rinsed in 70% ethanol, and then extensively washed with PBS. After the fly bodies were dissected in ice‐cold PBS and fixed with glutaraldehyde, 1‐mm posterior segment of the midgut was taken out and embedded in epoxy resin. The embedded midgut samples were sliced into the ultrathin section to be observed with a transmission electron microscope (JEM‐2100; Japanese Electronics Co., Ltd.). The mitochondria and microvilli of epithelial cell in the *Drosophila* midgut were observed and photographed for the extensive evaluation (Li‐Byarlay et al., 2016).

### 2.5 Quantitative real‐time PCR

The whole midguts were picked out as above described method to measure the expressing level of related genes by the quantitative real‐time PCR. Total RNA (20 midguts per sample) was extracted from whole midguts and was reverse‐transcribed into cDNA as the template for the examination. Primers were designed and synthesized by Wcgene Biotech, Shanghai, China. Using *rp49* as the reference gene, the calculation was performed using the 2^‐ΔΔCt^ method (Livak & Schmittgen, 2001). The primer sequences are shown in Table 1.

**TABLE 1:** qPCR primers

Gene name	Sequence 5’–3’	Annealing temp.
*AMPKα*	F:AGAGGTCTGCACCAAGTTCG R: GTTTATTTGGTTGGCCGCGT	60℃
*Atg1*	F:AAGGGCAGACAAGAGTCCAT R:GTTCTCCCGCTTCCTCCTTT	60℃
*Atg5*	F:ATATGCTTCCAGGCGGATCG R:AACCACACAGCTCCATCCTG	60℃
*Atg8a*	F:TCTAGCCACAGCAGTTAGCG R:TTGTGTAGAGTGACCGTGCG	60℃
*Relish*	F:GCATGGAACACATGGATCGC R:CTGATGGGAATGTGGGCTGT	60℃
*Dredd*	F:CATGGCCGGATCAAACCTGT R:AAGCAGAGGCCCACCTTTTG	61℃
*Fadd*	F:GAGCGGACGAACTATCGGAG R:CATTCTGGGAAGCTGGAGCA	60℃
*mTor*	F:AAAGAGCCAGACAGACGTGG R:CGACGCGAAGAGTTAAAGCG	60℃
*S6K*	F:CGCAGGACGAGATGATGGA R:TGGGATGGGTTGGTTGGT	60℃
*4E‐BP*	F:ACCCTCTACTCCACCACTCC R:GGAGTTTGGCTCAATGGGGA	60℃
*AttacinA*	F:GCATCCTAATCGTGGCCCT R:AGCGGGATTGGAGGTTAAGG	60℃
*CecropinC*	F:GCATTGGACAATCGGAAGCC R:GCGCGTTATCCTGGTAGAGT	60℃
*Defensin*	F:CTCGTGGCTATCGCTTTTGC R:CCACTTGGAGAGTAGGTCGC	60℃
*Diptericin*	F:CTCAATCTTCAGGGAGGCGG R:AGGTGCTTCCCACTTTCCAG	60℃
*Rp49*	F:AGGGTATCGACAACAGAGTG R:CACCAGGAACTTCTTGAATC	60℃

### 2.6 16S rDNA analysis

16S rDNA was used to analyze *Drosophila* intestinal microbial composition. DNA from whole midguts prepared as above described method was extracted using the E.Z.N.A. ®Stool DNA Kit (D4015; Omega, Inc.) according to the manufacturer's instructions. The total DNA was eluted in 50 μl of elution buffer and stored at –80°C until the measurement. PCR amplification was performed by targeting the 16S rRNA gene sequence (regions V6–V8), and libraries were prepared according to the guidelines provided by Illumina, provided by LC‐Bio. The amplified 16S rDNA fragments were then sequenced using the Illumina MiSeq platform (version 1.8.0) with the Microbiome Helper workflow. Chimeric sequences were filtered using Vsearch software (v. 2.3.4). Sequences with ≥ 97% similarity were assigned to the same operational taxonomic units (OTUs) by Vsearch (v. 2.3.4). Representative sequences were chosen for each OTU, and taxonomic data were then assigned to each representative sequence using the Ribosomal Database Project (RDP) classifier. The differences in the dominant species in different groups and multiple sequence alignment were conducted using MAFFT software (v. 7.310) to study the phylogenetic relationships of different OTUs.

### 2.7 Statistical analysis

All experiments were performed with at least three replicates. The significance of statistical differences was analyzed using the two‐tailed unpaired *t* test using GraphPad Prism 6 (Version No. 6.01; GraphPad Software). All data are expressed as mean ± *SD*. *p* < .05 was considered as statistical significance.

## 3 RESULTS

### 3.1 AOS improved the survival rate of SDS‐stimulated *Drosophila*

**FIGURE 1:**
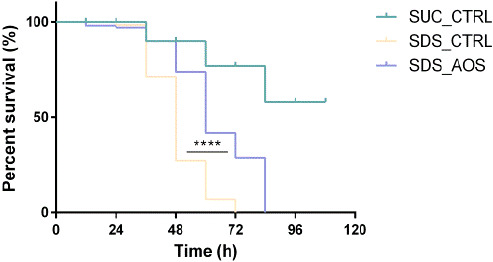
The survival rate of male *Drosophila* in the different treated groups. SUC_CTRL, SDS_CTRL, and SDS_AOS, respectively, represented the fly groups fed on 5% sucrose solution, 5% sucrose solution added with 0.6% SDS, and 5% sucrose solution including 0.6% SDS and 0.125% AOS. The log‐rank test revealed that AOS could significantly improve the survival rate of *Drosophila* suffered by SDS, ****p < .0001

To analyze the protective effect of AOS against the midgut damaged by SDS, we performed the survival experiments on *Drosophila*. All fruit flies in the SDS_AOS group and SDS_CTRL group died at the 84th and 72nd hour, respectively. However, the survival rate of the *Drosophila* in the SUC_CTRL group was still over 60% at the 84th hour (Figure 1). The average lifespan extension rates in the SDS_AOS groups were enhanced by 26.88% (58.87 hr ± 0.97 vs. 46.40 hr ± 2.4) in contrast to the SDS_CTRL groups. Moreover, the results of log‐rank analysis showed that the chi‐square values of SDS_AOS groups were 39.73 and the difference was statistically significant (****p < .0001) (Figure 1) between the SDS_AOS groups and the SDS_CTRL groups. These results revealed that the survival rate was significantly improved by AOS for SDS‐stimulated *Drosophila*.

### 3.2 AOS alleviated the microvilli damage of epithelial cells in *Drosophila* midgut by the SDS induction

**FIGURE 2:**
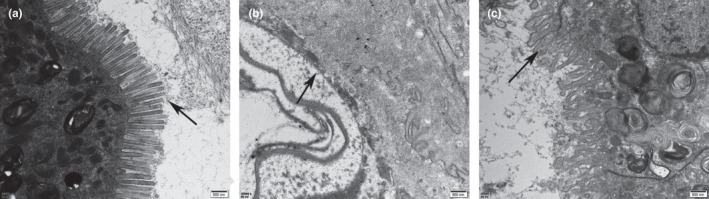
TEM images of the microvilli (arrow pointing) of *Drosophila* midguts. (a) The intestine from the flies fed on 5% sucrose solution (SUC_CTRL group); (b) the intestine from the flies fed on 5% sucrose solution added with 0.6% SDS (SDS_CTRL group); (c) the intestine from the flies fed on 5% sucrose solution including 0.6% SDS and 0.125% AOS (SDS_AOS group). Scale bar: 500 nm; magnification 20,000×

According to the examined results of transmission electron microscope (TEM), the microvilli of epithelial cells in the SUC_CTRL groups were neatly arranged, and no deletion was observed (Figure 2a). However, these microvilli were severely damaged and invisible on the intestinal epithelial cells in the SDS_CTRL group (Figure 2b). The microvilli of *Drosophila* intestinal epithelial cells in the SDS_AOS group were injured and in disorder to a certain extent, such as some microvilli truncature, but the damage was a little slight in comparison with those in the SDS_CTRL group (Figure 2c).

### 3.3 AOS alleviated mitochondrial damage of epithelial cells in *Drosophila* midgut by the SDS induction

**FIGURE 3:**
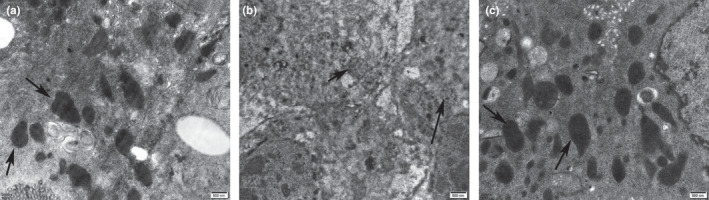
TEM images of mitochondrial (arrow pointing) of *Drosophila* midguts. (a) The intestine from the flies fed on 5% sucrose solution (SUC_CTRL group); (b) the intestine from the flies fed on 5% sucrose solution added with 0.6% SDS (SDS_CTRL group); (c) the intestine from the flies fed on 5% sucrose solution including 0.6% SDS and 0.125% AOS (SDS_AOS group). Scale bar: 500 nm; magnification 20,000×

As shown in Figure 3, the mitochondria in the fly midgut epithelial cells were intact in the SUC_CTRL group, and the cristae structure was clear with uniform dyeing of the matrix (Figure 3a). In the SDS_CTRL group, the mitochondria had swelling, vacuolization, and were stained lightly (Figure 3b). AOS reduced the SDS damage to mitochondria, which showed the intact morphology and distinct cristae structure similar to those in the SUC_CTRL group (Figure 3c).

### 3.4 AOS activated the expressions of antimicrobial peptides in the SDS‐stimulated intestines

**FIGURE 4:**
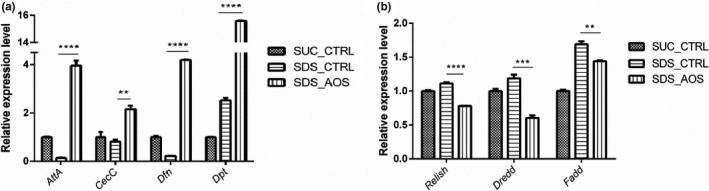
The effect of AOS on relative expression levels of AMPs (a) and IMD pathway (b) in the intestine of SDS‐stimulated *Drosophila*. SUC_CTRL, SDS_CTRL, and SDS_AOS, respectively, represented the fly groups fed on 5% sucrose solution, 5% sucrose solution added with 0.6% SDS, and 5% sucrose solution including 0.6% SDS and 0.125% AOS. The results are presented as the means ± *SEM*s (*n* = 3), and statistical comparisons were performed with *t* test; **p < .01, ***p < .001, and ****p < .0001

Antibacterial peptides (AMPs) are often used to represent the innate immune activity of *Drosophila*, and the immune deficiency (IMD) pathway, including the *Relish*, *Dredd,* and *Fadd* factors, is a critical regulator of antibacterial defenses in the fry guts and often directly regulates the AMP gene expression. Accordingly, the expressing levels of examined *Attacin A* (*AttA*), *Cecropin C* (*CecC*), *Defensin* (*Dfn*), and *Diptericin* (*Dpt*) had the obvious variance for the flies induced by SDS. Compared with SDS_CTRL group, the expression levels of the above four genes were significantly higher in the SDS_AOS group (*AttA*: 3.958 ± 0.21 vs. 0.1390 ± 0.018, *p* < .0001; *CecC*: 2.154 ± 0.15 vs. 0.8161 ± 0.076, *p* < .01; *Dfn*: 4.193 ± 0.014 vs. 0.2201 ± 0.0089, *p* < .0001; *Dpt*: 15.58 ± 0.047 vs. 2.523 ± 0.099, *p* < .0001, respectively in Figure 4a). The expression levels of *Relish*, *Dredd*, and *Fadd* were significantly downregulated by AOS supplementation (*Relish*: 0.7810 ± 0.0048 vs. 1.112 ± 0.020, *p* < .0001; *Dredd*: 0.6035 ± 0.038 vs. 1.188 ± 0.055, *p* < .001; *Fadd*: 1.441 ± 0.012 vs. 1.690 ± 0.044, *p* < .01, respectively, in Figure 4b). There were no consistent results of gene expressed levels between AMPs and IMD pathway.

### 3.5 AOS modified the gene expression levels of cell autophagy in the SDS‐stimulated intestines

**FIGURE 5:**
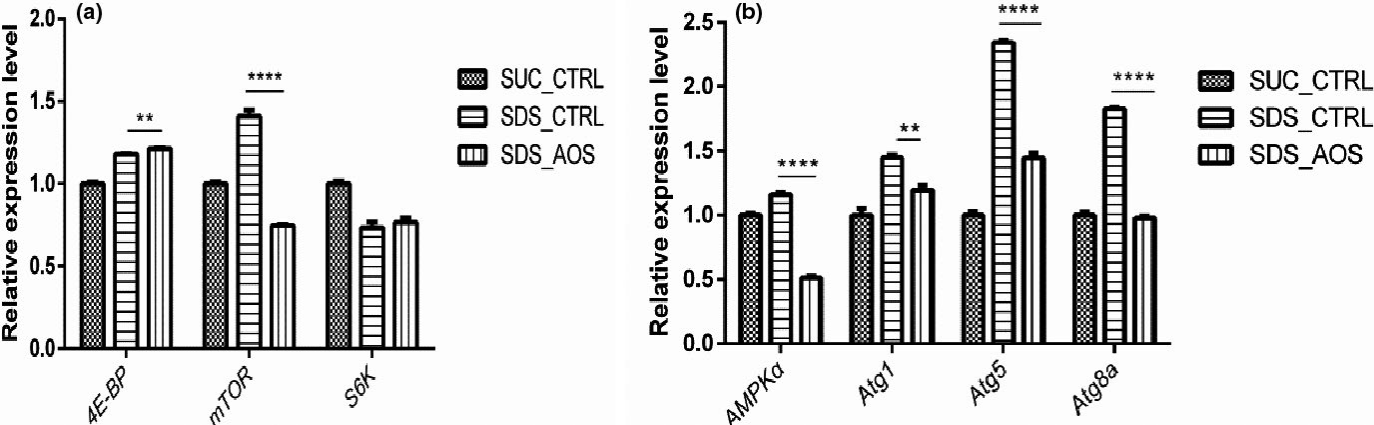
The effect of AOS on the relative expression levels of TOR pathway‐related gene (a) and cell autophagy (b). SUC_CTRL, SDS_CTRL, and SDS_AOS, respectively, represented the fly groups fed on 5% sucrose solution, 5% sucrose solution added with 0.6% SDS, and 5% sucrose solution including 0.6% SDS and 0.125% AOS. The results are presented as the means ± *SEM*s (*n* = 3), statistical comparisons were performed with *t* test; **p < .01 and ****p < .0001

To further study the effect of AOS on the midgut, we examined the expression levels of autophagy‐related genes including rapamycin (mTOR) signal pathway and autophagy process. All the detected genes showed the higher expressing levels in the SDS_CTRL group, but when AOSs were supplemented, the up‐expressing phenomenon had some variance for different genes (Figure 5a,b). Compared to the SDS_CTRL group, the expression levels of *4E‐BP* were upregulated (1.214 ± 0.0051 vs. 1.181 ± 0.0037, *p* < .01), the expression levels of *mTOR* were downregulated (0.7470 ± 0.0081 vs. 1.409 ± 0.0378, *p* < .0001), and the *S6K* expression had no significant difference (Figure 5a) in the SDS_AOS group. The expression levels of all detected autophagy process genes, *AMPKα, Atg1, Atg5,* and *Atg8a*, were inhibited by AOS in the SDS_AOS group in contrast to SDS_CTRL group (*AMPKα*: 0.5145 ± 0.011 vs. 1.162 ± 0.012, *p* <.0001; *Atg1*: 1.192 ± 0.040 vs. 1.449 ± 0.020, *p* < .01; *Atg5*: 1.444 ± 0.035 vs. 2.343 ± 0.016, *p* < .0001; *Atg8a*: 0.9787 ± 0.015 vs. 1.825 ± 0.014, *p* < .0001) (Figure 5b).

### 3.6 AOS improved the α‐diversity of midgut microbiota in SDS‐induced *Drosophila*

**FIGURE 6:**
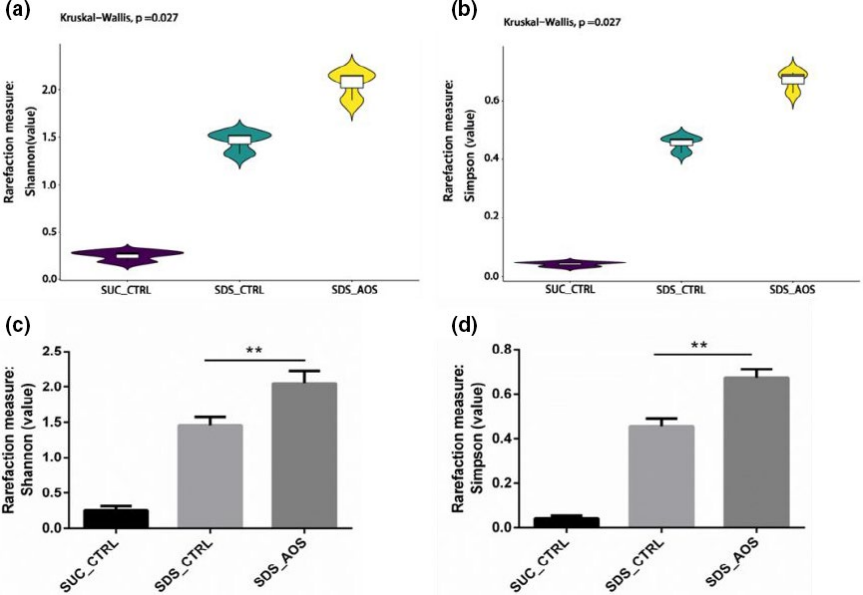
The effect of AOS on the α‐diversity of midgut microbiota in SDS‐induced *Drosophila*. SUC_CTRL, SDS_CTRL, and SDS_AOS, respectively, represented the fly groups fed on 5% sucrose solution, 5% sucrose solution added with 0.6% SDS, and 5% sucrose solution including 0.6% SDS and 0.125% AOS. The *p*‐value was obtained by the Kruskal–Wallis test of all groups, and *p* < .05 indicated that the three groups were different samples (a, b). The results are presented as the means ± SEMs (*n* = 3), and the statistical comparisons were performed with *t* test; **p < .01 (c, d)

The Shannon index and Simpson index were used to analyze α‐diversity index of intestinal microbiota. The result of the Kruskal–Wallis was *p* =.027, indicating that the three group microbiota came from different samples (Figure 6a,b), and the Shannon and Simpson indexes of the SDS_CTRL and SDS_AOS groups had a significant improvement of intestinal microbiota α‐diversity for the flies induced by the SDS in contrast to those of the SUC_CTRL group (Figure 6a,b). The *t* test results showed that the Shannon and Simpson indexes of intestinal microbiota in the SDS_AOS groups were higher in contrast to those in the SDS_CTRL groups (*p* < .001) (Figure 6c,d), which showed the better diversity with the AOS supplement.

### 3.7 AOS changed the β‐diversity of midgut microbiota in SDS‐induced *Drosophila*

**FIGURE 7:**
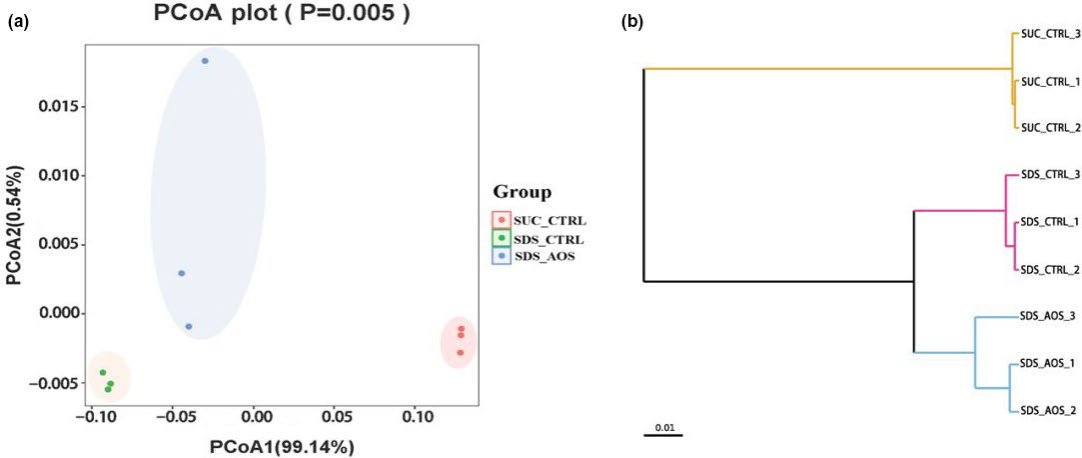
The effect of AOS on the β‐diversity of midgut microbiota in SDS‐induced *Drosophila*. The same color dots of PCoA (a) came from the samples in one group, and the branching in the UPGMA (b) based on weighted UniFrac distance showed homologous. SUC_CTRL, SDS_CTRL, and SDS_AOS, respectively, represented the fly groups fed on 5% sucrose solution, 5% sucrose solution added with 0.6% SDS, and 5% sucrose solution including 0.6% SDS and 0.125% AOS

The diversity of species between different environmental communities can be indicated by β‐diversity. Principal coordinate analysis (PCoA) and unweighted pair group method with arithmetic mean (UPGMA) based on weighted UniFrac distance had been used to evaluate β‐diversity. The results showed that there was significant difference between the microbial species diversities in the *Drosophila* intestinal microbita before and after SDS stimulation according to the distribution position of the same color dots (Figure 7a), and the dots were close to PCoA_2_ lower quadrant in the SDS_AOS (blue dots) and SUC_CTRL (red dots) groups, but those were located in the broader area in the SDS_CTRL group. In addition, compared with that of between the SUC_CTRL group and SDS‐induced groups, there was the higher homology of microbial species in the two SDS‐induced groups (Figure 7b).

### 3.8 AOS improved the midgut microbial composition in the SDS‐induced *Drosophila*

**FIGURE 8:**
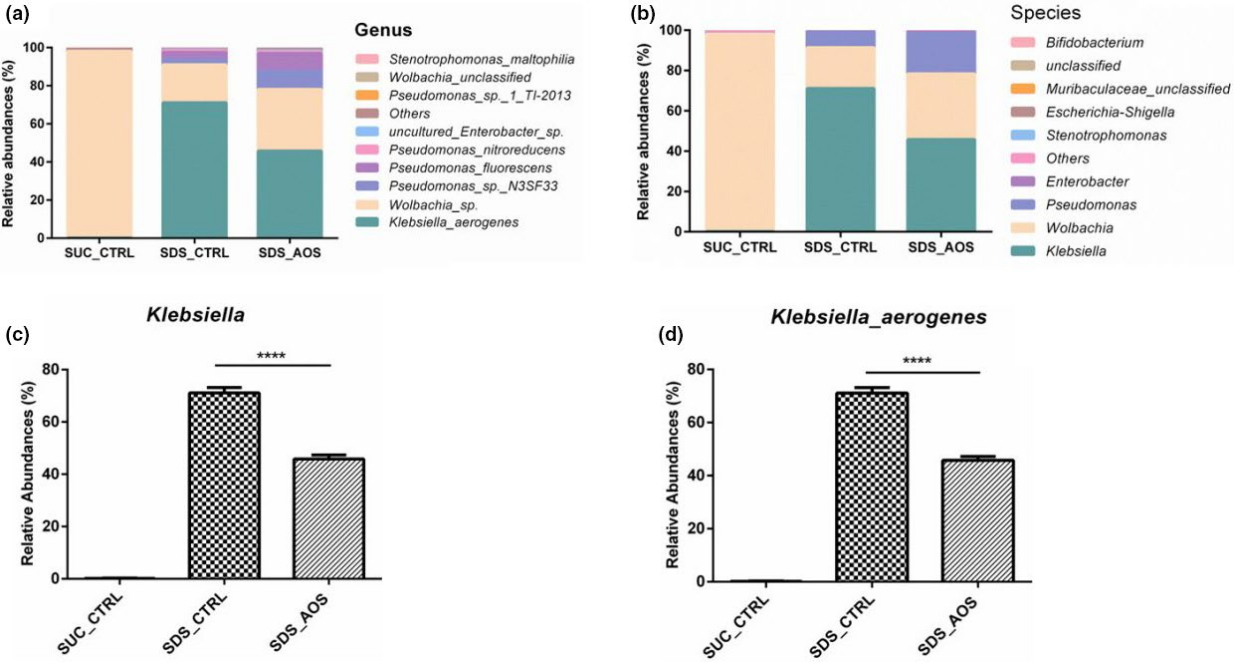
The effect of AOS on midgut microbial composition in SDS‐induced *Drosophila*. Relative abundance of midgut microbes at the genus (a) or species (b) level. The abundance of *Klebsiella* or *Klebsiella_aerogenes* was compared between the SDS_CTRL and SDS_AOS groups (c, d). SUC_CTRL, SDS_CTRL, and SDS_AOS, respectively, represented the fly groups fed on 5% sucrose solution, 5% sucrose solution added with 0.6% SDS, and 5% sucrose solution including 0.6% SDS and 0.125% AOS

**FIGURE 9:**
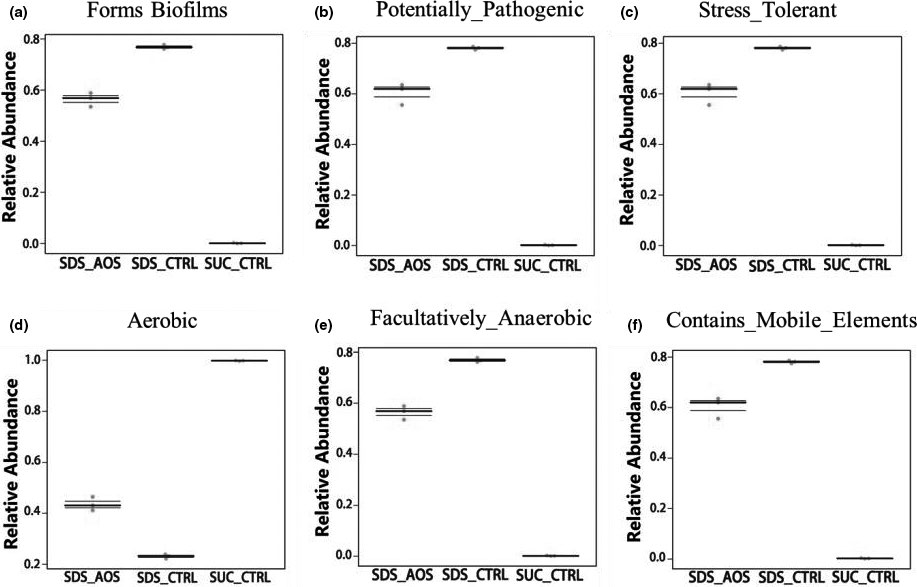
The prediction of related bacterial phenotypes of midgut microbiota in SDS‐induced *Drosophila,* including formation of biofilms (a), potentially pathogenic (b), stress‐tolerant (c), aerobic (d), facultatively anaerobic (e), and containing mobile elements (f). SUC_CTRL, SDS_CTRL, and SDS_AOS, respectively, represented the fly groups fed on 5% sucrose solution, 5% sucrose solution added with 0.6% SDS, and 5% sucrose solution including 0.6% SDS and 0.125% AOS

Midgut microbes of *Drosophila,* which were not treated with SDS to induce inflammation, mainly consisted of *Wolbachia* (Figure 8a,b). However, at the genus level, midgut microbiota changed from *Wolbachia* to *Klebsiella* after inflammation by SDS (Figure 9a). In addition, at the species level, *Klebsiella aerogenes* was the dominant bacteria in the inflammation groups (Figure 8b). Compared to the SDS_CTRL groups, the abundance of *Klebsiella* significantly dropped (45.90 ± 0.89 vs. 71.13 ± 1.17, *p* <.0001) (Figure 8c), and the abundance of *Klebsiella aerogenes* significantly decreased (45.82 ± 0.88 vs. 71.08 ± 1.18, *p* < .0001) in the SDS_AOS groups (Figure 8d).

### 3.9 Prediction of phenotypes of gut microbiota in *Drosophila*

Multiple host factors were influenced by intestinal microbes, including immunology, metabolism, and oxidative stress. Therefore, the function knowledge of intestinal microbes is extremely valuable to help disease testing, mechanism exploring, and target treating. According to the change in microbial groups mentioned above, the results showed that an abundance of bacteria, which promoted the formation of biofilms, was lower in the noninflammation groups (SUC_CTRL) than in the inflammation groups (SDS_AOS and SDS_CTRL), and these bacteria were decreased by AOS treatment in the inflammation groups (Figure 9a). The other microbial phenotypes, such as potentially pathogenic (Figure 9b), stress‐tolerant (Figure 9c), aerobic (Figure 9d), facultative anaerobic (Figure 9e), and containing mobile elements (Figure 9f), had the same trends. According to the above results, we think that AOS inhibited the relative abundance of microbes in midgut after inflammation induction by SDS. The abundance of aerobic bacteria was higher in the noninflammation groups and significantly dropped after the SDS induction. However, compared to the SDS_CTRL groups, the abundance of aerobic bacteria increased after the AOS supplement. Therefore, we think that AOS could efficiently inhibit the decreasing trend of aerobic bacteria after inflammation.

## 4 DISCUSSION

The gastrointestinal tract forms the largest and most important immune epithelial barrier that protects the organism against external dangers posed by ingested harmful pathogens (Capo et al., 2019). When the pathogen infection occurs, its causing inflammation often results in intestinal mucosal damage and barrier function impairment and the variation of micriobial composition. AOSs, as a kind of oligosaccharide of better water solubility and easier absorption, have exhibited the biological activities, such as antioxidation, antitumor, and immune activation (Higashimura et al., 2013). Additionally, our previous study indicated that AOS could significantly improve the average life expectancy and maximal lifespan of male flies by increasing antioxidant capacity and intestinal immunity, regulating the intestinal microbiota (Ma et al., 2019). *Drosophila* has a similar intestine anatomical structure and physiological function to humans, both of which come from the endothelial tissue (Pitsouli et al., 2009; Tepass & Hartenstein, 1994). In the study, survival assay and the ultrastructure of intestinal cells were firstly evaluated to examine the anti‐inflammatory effects of AOS.

The results of survival assay showed that the suitable dose of AOS could improve the survival rate of the SDS‐induced *Drosophila*. The transmission electron microscope results showed SDS induction reduced the length and uniformity of microvilli in the male *Drosophila* intestine, and AOS alleviated this damage of microvilli and mitochondria in the epithelial cells. According to the references, the extended part of enterocyte cytoplasm and intestinal stem cells is microvilli in *Drosophila*, which protects the intestine from microbes by comprising the brush edge and secreting mucus (Crosnier et al., 2006). Moreover, intestinal epithelial cell microvilli are essential for the balance of epithelial transport, and the morphology and length of microvilli directly affect the intestinal absorption function (Postema et al., 2019). The protective function of AOS supplement in the food could improve the nutrient absorption and reduce the harmful pathogen infection through the microvilli. Additionally, the mitochondrial dysfunction was one cellular hallmarks of aging, each of which has been proposed to contribute to age‐related health decline (Aparicio et al., 2019). The intact and dynamic mitochondria would provide the more energetic metabolism. Therefore, the above results showed that AOS could improve the ultrastructure function, which could enhance the absorbent ratio for the nutrient ingredient leading to higher survival rate.

Antimicrobial peptides expressed in the intestine can eliminate foreign pathogens (Tzou et al., 2000), promote the proliferation of profitable microbes that came from the environment, and often used as readouts to monitor the activity of these immune pathways (Hanson & Lemaitre, 2020). To explore the protection of AOS to the *Drosophila* intestine, we analyzed four important genes (*AttA*, *CecC*, *Dfn,* and *Dpt)* by qPCR, all of which were involved in AMP formation. Different from *AttA* and *Dpt*, which mainly have an antibacterial function, *CecC* and *Dfn* also function to inhibit fungi (Kragol et al., 2001; Tzou et al., 2002). The results of the assay showed that AOS‐supplemented food significantly increased the expression of the above genes. Hence, AOS activated the expression of AMPs in the *Drosophila* intestine after inflammation.

The IMD pathway is important for the defense of bacteria in *Drosophila*, and *IMD* expression in the intestine influences the genes involved in the development of the body and metabolism (Erkosar et al., 2014). Additionally, several researches showed that immune deficiency (IMD) could regulate antimicrobial peptides and had other immunology functions (Hanson & Lemaitre, 2020). The activation of NF‐κB depends on *Dredd*, a key cystine enzyme in the IMD pathway, which is also important in the activation of the JNK pathway (Guntermann & Foley, 2011). Fas‐associated death domain (*FADD*) could participate in the activation of the NF‐κB pathway and in apoptotic signal transport (Zhao et al., 2020). Under normal conditions, the activated Relish, another important protein in the IMD pathway, can promote the transcription of broad‐spectrum antimicrobial peptides (Zhao et al., 2020). In the experiment, the expressions of *Dredd*, *FADD,* and *Relish* were inhibited in SDS‐stimulated *Drosophila* intestine by AOS supplementation, which indicated that higher AMP expression level was not correlative with the IMD pathway.

Autophagy participates in the decomposition of damaged particles in cells, and recycles them to be used, which provides the basis for helping the biosynthesis reaction and energy production (Maruzs et al., 2019). When the intestine is subjected to injury, excessive autophagy occurs in the induced area. Numerous lines of evidence indicate that inflammation in immunity is linked to autophagy (Karunakaran et al., 2019). AMP‐activated protein kinase (*AMPK*) is a crucial energy sensor in cells and involve in autophagy by direct phosphorylation of the UNC‐51‐like kinase 1 (ULK1) (Egan et al., 2011). In this study, the results showed that the expression of autophagy‐associated genes significantly decreased after AOS supplementation. As the degree of inflammation in SDS_AOS groups was lower than SDS_CTRL groups, the inhibited cell autophagy might show that AOS relieved the damage caused by inflammation in the intestine.

The target of rapamycin (TOR) pathway is an important pathway controlling the lifespan of *Drosophila*. Mammal target of rapamycin (mTOR) signal can participate in the aging process in complex ways, which is associated with autophagy and cell stress (Kapahi et al., 2004). *S6K* is another effector in the TOR pathway, and its decrease or loss may extend the age of *Drosophila* (Toshniwal et al., 2019). *4E‐BP* could prolong the lifespan by enhancing the vitality of mitochondria (Zid et al., 2009). Results showed that the expression level of *mTOR* decreased and *4E‐BP* increased in the experimental group. These above results indicated that AOS supplementation in the basic diet of SDS‐induced *Drosophila* could alleviate intestinal inflammation, and its reason might be related to the downregulation of *mTOR* and the upregulation of *4E‐BP,* which was consistent with the reducing mitochondria injures. And the inhibiting cell autophagy of AOS might be related to the downregulation of AMPK and *mTOR* and upregulation of *4E‐BP*. According to the above results, we confirmed that inflammation stress would be alleviated by AOS via regulating *TOR* and *AMPK* pathways to reduce excessive cell autophagy.

The composition of microbes is critical to maintaining the health of the body, which influences the completeness of the intestinal barrier and steady state of the intestine (Clark et al., 2015). Hence, we analyzed the intestinal microbes of different groups of SDS‐induced *Drosophila* by a 16S rDNA test. The results showed that α‐diversity of intestinal microbiota was higher in the SDS_AOS groups than in the SDS_CTRL groups, which could be further confirmed by microbial composition. The α‐diversity improvement of intestinal microbiota could be the results of the intestinal damage by SDS induction, which was conducive to bacterial reproduction. The variation of microbiota β‐diversity showed that SDS had the significant effect on the intestinal microbial composition, but AOS could mitigate the radical alteration. At the genus level, the major detected bacterium was *Klebsiella* in both the SDS_AOS and SDS_CTRL groups, but the abundance of *Klebsiella* was significantly lower in the SDS_AOS group. At the species level, the dominant bacterium was proved to be *Klebsiella aerogenes*. Studies showed that *Klebsiella aerogenes* is a Gram‐negative facultative anaerobic bacteria and belongs to Enterobacteriaceae, which is associated with infections including pneumonia, urinary tract infection, and wound infection (Malek et al., 2019). The declining abundance of *Klebsiella aerogenes* in SDS_AOS groups indicated that AOS could alleviate inflammation by regulating the composition of intestinal microbes.

The formation of biofilms could help the microbes to be resistant to drugs, which could also protect their proliferation. Damaging the biofilms of bacteria is an effective method to treat bacterial infection (Rabin et al., 2015). The biofilm formed on medical devices by *Klebsiella pneumoniae* is an important cause of hospital infections (Murphy & Clegg, 2012). The existence of mobile elements could enhance the pathogenicity and resistance to drugs or antibiotics of bacteria (Zhang et al., 2018). Other research has shown that mobile elements of *Klebsiella aeruginosa* could play a major part in the expression of drug resistance. In this study, the authors predicted that a certain amount of AOS could effectively decrease the relative degree of mobile elements and biofilm formation of intestinal microbes in the inflammation of SDS‐induced *Drosophila*, which inhibited the proliferation, pathogenicity, and drug resistance of harmful bacteria. Prediction results showed that AOS significantly decreased the pathogenicity of intestinal microbes and suppressed the stress reaction by SDS. The abundance of aerobic and facultative anaerobic bacteria between the SDS_AOS and SDS_CTRL groups was significantly different, which also provided another testimony about AOS regulating the microbial composition to ameliorate the inflammation of the SDS‐induced *Drosophila* intestine.

## 5 CONCLUSION

Agar oligosaccharide significantly improved the survival rate of male *Drosophila* by decreasing the damage of epithelial cells in the intestine by SDS induction, in which mechanism could include improving the immune capacity by upregulating the AMP expression, suppressing the excessive autophagy by activating the TOR and AMPK pathways, and reducing the inflammatory stress by regulating the intestinal microflora.

## CONFLICT OF INTEREST

The authors declare that they have no conflicts of interest.

## ETHICAL APPROVAL

This study does not involve any human or animal testing.
